# Metabolomics to understand metabolic regulation underpinning fruit ripening, development, and quality

**DOI:** 10.1093/jxb/erad384

**Published:** 2023-10-21

**Authors:** Félix Juan Martínez-Rivas, Alisdair R Fernie

**Affiliations:** Centro de Biotecnología y Genómica de Plantas, Universidad Politécnica de Madrid (UPM) – Instituto Nacional de Investigación y Tecnología Agraria y Alimentaria (INIA-CSIC), Madrid, Spain; Departamento de Bioquímica y Biología Molecular, Universidad de Córdoba, Edificio Severo Ochoa, Campus de Rabanales, E-14014, Córdoba, Spain; Max-Planck-Institute of Molecular Plant Physiology, Am Mühlenberg 1, 14476 Potsdam-Golm, Germany; RIKEN Center for Sustainable Resource Science, Japan

**Keywords:** Development, flavor, nutrition, primary metabolite, ripening, secondary metabolite

## Abstract

Classically fruit ripening and development was studied using genetic approaches, with understanding of metabolic changes that occurred in concert largely focused on a handful of metabolites including sugars, organic acids, cell wall components, and phytohormones. The advent and widespread application of metabolomics has, however, led to far greater understanding of metabolic components that play a crucial role not only in this process but also in influencing the organoleptic and nutritive properties of the fruits. Here we review how the study of natural variation, mutants, transgenics, and gene-edited fruits has led to a considerable increase in our understanding of these aspects. We focus on fleshy fruits such as tomato but also review berries, receptacle fruits, and stone-bearing fruits. Finally, we offer a perspective as to how comparative analyses and machine learning will likely further improve our comprehension of the functional importance of various metabolites in the future.

## Teaser

Fruits undergo dramatic physiological changes through their developmental and ripening phases, highlighting the metabolic changes linked to flavor. The use of different metabolic platforms helps in understanding these changes.

## Introduction

The role of metabolites in fruit ripening and development has long been recognized, particularly in the model fruit crop tomato. Fruit development is a highly complex process that has received considerable research attention with the vast majority of studies being concentrated on hormonal regulation (Lanahan *et al.*, 1994; Adams-Phillips *et al.*, 2004; Barry and Giovannoni, 2006), pigmentation (Giuliano *et al.*, 1993; Fraser *et al.*, 1994; Ronen *et al.*, 2000), and sugar (Yelle *et al.*, 1991; Klann *et al.*, 1996; Fridman *et al.*, 2004), cell wall (Osorio *et al.*, 2020; D. Wang *et al.*, 2018), and organic acid metabolism (Carrari *et al.*, 2006; Centeno *et al.*, 2011). Early studies describing the roles of metabolites in these processes depended on looking either at correlations between the levels of select metabolites and the ripening process (Schaffer and Petreikov, 1997), or at the metabolic characterization of ripening mutants (Osorio *et al.*, 2011). These have recently been extended in scope following the advent and widespread adoption of metabolomics (Carrari *et al.*, 2006; Osorio *et al.*, 2011; Nardozza *et al.*, 2013; Klie *et al.*, 2014). However, more recently such efforts have been complemented by quantitative trait locus (QTL)- or genome wide association study (GWAS)-based approaches (Causse *et al.*, 2004; Tieman *et al.*, 2017; Zemach *et al.*, 2023), as well as reverse genetic strategies employing transgenic and latterly gene-editing approaches (Karlova *et al.*, 2011; Lee *et al.*, 2012; Li *et al.*, 2023; Yang *et al.*, 2023). As we will detail below such forward and targeted reverse-genetic strategies have greatly enhanced our mechanistic understanding of these processes.

The role of metabolites in fruit quality is similarly well documented (Klee and Tieman, 2018). Indeed, studies on the metabolic factors that are important for both taste and nutrition are widespread. Much of this work has also been carried out in tomato with particularly notable studies being those of the Klee group, defining the genetics that underlie the taste of tomato fruit (Tieman *et al.*, 2017; Klee and Tieman, 2018), and those of the Martin group, evaluating nutritionally important components of tomato (Zhang *et al.*, 2015; Martin and Li, 2017; Butelli *et al.*, 2019; Li *et al.*, 2022). However, work has extended well beyond this species, with studies recently focusing on the change of crop chemical composition during the domestication of a wide range of species (Alseekh *et al.*, 2021b) including the fruit species tomato (Zhu *et al.*, 2018), melon (Ren *et al.*, 2021; Yuan *et al.*, 2023), jejube (Zhang *et al.*, 2022), peach (Cao *et al.*, 2022), pummelo (Zheng *et al.*, 2023), apple (Lin *et al.*, 2023), citrus (Wang *et al.*, 2017), and watermelon (Yuan *et al.*, 2023). These changes, whilst very much species-specific, were general in that they frequently reduced the accumulation of bitter-tasting substances (Alseekh *et al.*, 2021b). Besides this, other studies trying to directly link the metabolomic status of fruits with their taste or nutritional compounds have been published in a range of species including citrus (Lin *et al.*, 2015), kiwi (Wang *et al.*, 2022), grapevine (Mattivi *et al.*, 2006; Cuadros-Inostroza *et al.*, 2016), peach (Lombardo *et al.*, 2011), pear (Oikawa *et al.*, 2015), melon (Moing *et al.*, 2011), and strawberry (Fait *et al.*, 2008).

Given that myriad reviews detail technical aspects of metabolomics, we will not cover these in any detail here, but refer the readers to the articles by Perez de Souza *et al.* (2017, 2019, 2021) and Alseekh *et al.* (2021a), who extensively cover machine-based aspects of this topic, and by Kopka *et al.* (2004) and Tohge *et al.* (2011), who cover sampling-based aspects. However, we will document in brief the major technical and computational approaches adopted in these studies. Regarding the technological approaches, essentially three different platforms are used—nuclear magnetic resonance (NMR) spectroscopy, gas chromatography–mass spectrometry (GC-MS), and liquid chromatography–mass spectrometry (LC-MS) (Table 1) (Obata and Fernie, 2012). Until recently GC-MS was the most widely used technique for plant metabolomics research to date. The crucial advantage of GC-MS is that it has long been used for metabolite profiling, and thus there are stable protocols for machine setup and maintenance and for chromatogram evaluation and interpretation (Fernie *et al.*, 2004; Halket *et al.*, 2005; Lisec *et al.*, 2006). The robustness of the protocol means that libraries of retention time and mass spectra data for standard compounds can be shared among laboratories and several metabolite databases are available that aid in peak annotation (Perez de Souza *et al.*, 2017). Additionally, the short running time and relatively low running costs are also strong advantages of GC-MS. However, its use is limited to thermally stable volatile compounds, rendering the analysis of high molecular weight compounds (larger than 1 kDa) difficult. That said a wide range of derivatization agents can be employed that render small non-volatile compounds volatile. These have, however, been extensively reviewed previously (Halket *et al.*, 2005), and we will therefore not detail them here. While GC has a limitation due to volatilization of compounds, LC does not require prior sample treatment and separates the components in a liquid phase. The choice of columns, including reversed phase, ion exchange, and hydrophobic interaction columns, provides for separation of metabolites based on different chemical properties. Therefore, LC has the potential to analyse a wide variety of metabolites in plants. The recent development of ultra-performance liquid chromatography and high-resolution MS rendered the technique yet more powerful due to the additional higher resolution, sensitivity, and throughput than conventional high-performance liquid chromatography (Alseekh and Fernie, 2018; Perez de Souza *et al.*, 2021). The versatility conferred by these improvements has led to LC-MS becoming the method of choice for metabolomics. However, the flip side of this is that it causes difficulty in establishing large mass spectral libraries for peak identification because of the instrument-type-dependent retention time and mass spectra (Horai *et al.*, 2010), and forces each research group to create its own ‘in-house’ LC-MS reference library. That said, there are a number of websites that aid in mass-spectral analyses (Perez de Souza *et al.*, 2017), and recent recommendations for metabolite reporting (Alseekh *et al.*, 2021a) should improve the transparency of the methods used by researchers. NMR spectroscopy offers an entirely different analytical technique, being based on atomic interaction. Atoms in a molecule give a specific spectrum of radiation that can be used for identification and quantification of metabolites within a complex biological sample (Obata and Fernie, 2012). The sensitivity of this method is much lower than that of MS-based techniques but the structural information content, reproducibility, and quantitative aspects can be superior (Fernie *et al.*, 2004). Moreover, for isotope labelling, NMR has the specific advantage of providing facile access to atomic-level labelling, which is highly laborious in the case of MS methods yet can be essential in flux estimation (see for example Fernie *et al.*, 2001). However, the number of compounds that can be detected in a single analysis is dramatically below that achievable by MS-based methods (Fernie *et al.*, 2004).

**Table 1.** Comparison of the scope and properties of the most used metabolomics platforms

Method	Measured compounds	Advantages	Disadvantages
Non-derivatization-based GC-MS	Volatile compounds	Simple sample preparation	Low number metabolites detected
Derivatization-based GC-MS	Small (<500 Da) polar metabolites (including amino acids, sugars, organic acids)	Highly reproducible High sensitivity Free libraries accessible	Relatively complex sample preparation
LC-MS	Large (>500) non-polar metabolites (pigments, hormones, lipids, phosphorylated metabolites, secondary metabolites such as polyphenols, alkaloids, and glucosinolates)	Simple sample preparation High sensitivity Novel compound identification	Resolution Reproducibility Free libraries and availability of reference compounds
CE-MS	Ionic metabolites	Simple sample preparation High sensitivity High resolution	Lack of reference libraries Difficult to run at high throughput.
NMR	Abundant metabolites	Atomic structure determination Simple sample preparation	Low sensitivity Cost of running Amount of sample needed

CE-MS, capillary electrophoresis–mass spectrometry; GC-MS, gas chromatography–mass spectrometry; LC-MS, liquid chromatography–mass spectrometry; NMR, nuclear magnetic resonance.

The specific method of detection is, however, not the focus of our current review, since it is our view that once properly validated the biological meaning of the metabolite shifts observed are of greater value. In this vein, for the purposes of this review, we will mainly discuss (i) seed-bearing fleshy fruits, (ii) stone-bearing fruits, and (iii) berries, which are best characterized in the literature. Nonetheless, we refer the readers to the review of Shiratake and Suzuki (2016), which covers omic studies in citrus, grape, and *Rosaceae* as well as the literature cited above that contain species that are not included in this review (Shiratake and Suzuki, 2016). In each case we will discuss what has been learnt regarding the role of metabolites in their development and/or quality from the study of cultivated and wild species, of classical mutants, and using transgenic and gene-editing approaches. We will end this review with a synthesis of commonalities and differences between species within and between the fruit classes given above as well as discussing how metabolomics is currently being adopted within multi-omics approaches to provide a higher resolution definition of aspects of metabolic regulation that will likely prove highly informative in selective breeding tools for improving fruit quality. These discussions will also include the use of metabolomics data in genomic prediction and machine learning applications, which may revolutionize breeding for altered (fruit) crops in the coming decades.

## Seed-bearing fleshy fruits

### Ripening and post-ripening based metabolic shifts

Most work on fruit ripening and quality over the last decades has focused on seed-bearing fleshy fruits with this work being predominantly carried out in tomato. This species has several advantages as a model system including a relatively small genome size, a relatively short life cycle and routine transient and stable transformation protocols as well as being agronomically important in its own right. Given that the vast majority of research has focused on this species we will utilize it as a case study for seed-bearing fruits. We would, however, like to acknowledge that considerable studies have been carried out in other species including pepper (Kim *et al.*, 2020), kiwi (Fernie and Alseekh, 2022b; Wang *et al.*, 2022), and melon (Moing *et al.*, 2011). Whilst these studies reveal much commonality in the changes that occur on ripening, there are also considerable differences particularly between climacteric fruits such as tomato and kiwi, which experience an ethylene induced respiratory burst on ripening, and non-climacteric fruits such as pepper and oranges, which do not (we return to this point below).

Early insights into the metabolic changes occurring on tomato ripening came from commercial ripening mutants that had been generated as a route to extend tomato shelf-life. For example, mutation at the *rin* locus affects all aspects of the tomato fruit ripening process, being characterized by its complete lack of ripening (Giovannoni *et al.*, 2017), with its molecular cloning revealing tandem MADS box genes of which only one was necessary for ripening (Vrebalov *et al.*, 2002). A phenotypically similar mutant with regard to responsiveness to ethylene was named *nor* (non-ripening) (Lincoln and Fischer, 1988). Further genetic analysis showed that these mutations reside in different unlinked loci (Giovannoni *et al.*, 1995). However, whilst it is known that *nor* encodes a transcription factor (TF), it is not as well characterized as *rin*. Other ethylene-insensitive ripening mutants of note are *Never ripe* (*Nr*) (Lanahan *et al.*, 1994), *Green ripe* (*GR*) (Jarret *et al.*, 1984), and *Never ripe 2* (*NR2*) (Kerr, 1982), whilst *Colourless non-ripening* (*Cnr*) encodes an SPNP TF that is also a necessary regulator of ripening (Manning *et al.*, 2006). Furthermore, the high-pigment mutants *de-etiolated* and *damaged DNA-binding proteins 1* influence the role of light in ripening (Enfissi *et al.*, 2010; Wang *et al.*, 2019), with other vital TFs in the process including FRUITFUL1, SlNAC4, APELETTA2, GOLDEN-LIKE KINASE2, and WD40 (Karlova *et al.*, 2011; Bemer *et al.*, 2012; Powell *et al.*, 2012; Zhu *et al.*, 2022).

Early studies on the long shelf-life mutants revealed that they hindered the change in color and also softening on ripening (Bird and Ray, 1991; Pecker *et al.*, 1992; Sakurai and Nevins, 1993; Scheible and Pauly, 2004; Cazzonelli and Pogson, 2010; Yang *et al.*, 2017; N. Wang *et al.*, 2018). More recently, integrated chromatin immunoprecipitation and transcriptome analysis revealed that these mutants affect genes involved in ethylene biosynthesis, pigment formation, cell wall degradation, volatile production, and phenylpropanoid metabolism (Fujisawa *et al.*, 2012; Irfan *et al.*, 2016). An early detailed GC-MS-based study of primary metabolite compositional changes on ripening of wild type tomato (Carrari *et al.*, 2006) revealed that changes in levels of a broad range of sugars mirrored those already observed for the major sugars of the fruit (Suc, Glc, and Fru) or the enzymes involved in their interconversion (see Robinson *et al.*, 1988; Miron and Schaffer, 1991; Schaffer and Petreikov, 1997). Moreover, this study revealed patterns of change in organic acid levels that mirrored changes in the activities of the enzymes of the TCA cycle (Jeffery *et al.*, 1986) or were consistent with changes previously recorded for the major fruit acids (malate and citrate) (Davies, 1966). Similarly, the very large increases in many amino acids mirror those reported almost 40 years ago for glutamate (Grierson *et al.*, 1985); however, it is noteworthy that many amino acid levels also decreased during ripening (Carrari *et al.*, 2006). Intriguingly, the study of Carrari *et al.* (2006) carried out transcriptomics in parallel to metabolomics and visualization of correlations between the metabolites measured and known determinants of tomato ripening, including genes associated with cell wall, ascorbate, and carbohydrate metabolism as well as TFs associated with ripening. Genes associated with the ethylene pathway exhibited a striking correlation with a subset of the organic acids, such as TCA cycle intermediates, gluconate, quinate, and shikimate, among others. Indeed, this work promoted the study of Centeno *et al.* (2011), who performed reverse genetic testing of the importance of organic acids in ripening by using transgenic tomato that was deficient in the expression of malate dehydrogenase or fumarase. These plants were characterized by accelerated or decelerated ripening, manifesting the importance of TCA cycle intermediates for the regulation of ripening, as later confirmed in Osorio *et al.* (2011) and discussed in the next paragraph. Alongside, they also presented differences in content of total soluble solids and in post-harvest decay and susceptibility to disease (Centeno *et al.*, 2011).

Metabolomics has, over the last two decades, additionally been applied to characterize the metabolic changes occurring in the majority of these ripening mutants. The study of Osorio *et al.* (2011) profiled the primary metabolic changes that occurred in *nor*, *rin*, and *Nr*, alongside the transcript and protein changes across a dense time course similar to that of Carrari *et al.* (2006) described above. It reported that the reduction of malate reported in the wild type was much diminished in *rin* and *Nr* but unaltered in *nor*. Moreover, *rin* and *Nr* were characterized by enhanced sucrose degradation whilst all three mutants exhibited decreased levels of aromatic amino acids. Decreased aromatic and branched chain amino acids appears to be a common feature of ripening mutants, also being observed in *Apelata2* (Karlova *et al.*, 2011) and *WD40* (Zhu *et al.*, 2022), two different ripening mutants, probably reflecting the use of these metabolites as alternative respiratory substrates that support energy metabolism during the climacteric ripening.

**Fig. 1.**
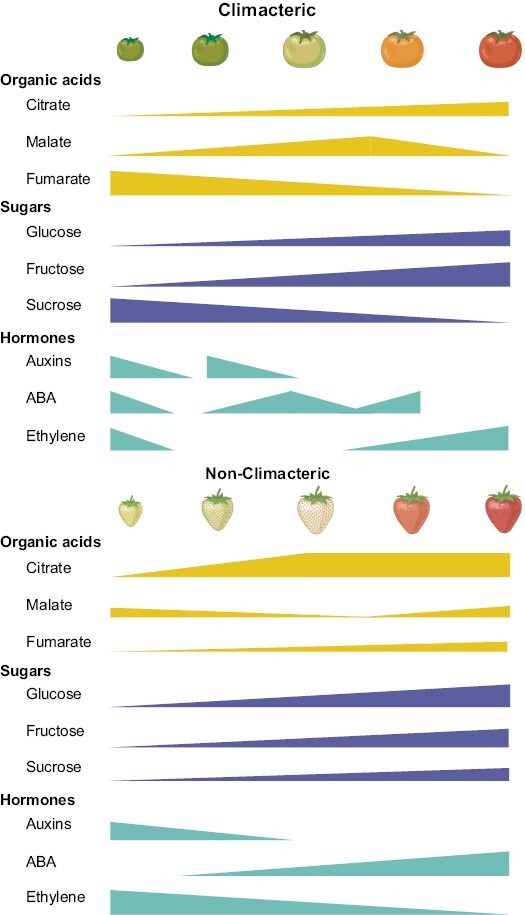
Metabolic changes in organic acids, sugars, and hormones in two examples, climacteric *Solanum lycopersicum* and non-climacteric *Fragaria* × *ananassa.* Based on Batista-Silva *et al.* (2018). Created with BioRender.com.

Osorio *et al.* next compared tomato ripening, which as we mentioned above is climacteric, with that of pepper, which is not. Intriguingly, they found that the metabolic shifts that each species underwent had little overlap between them. In fact, the only metabolites that presented a similar pattern between tomato and pepper are the sugars fructose and glucose (Osorio *et al.*, 2012). More studies have compared the metabolic shifts between climacteric and non-climacteric species. Given the availability of massive amounts of metabolomic data, comparative analysis of different fruit types will aid in our understanding of the functional importance of metabolites in different species. Three studies are particularly pertinent in this respect. In the first, study of different species was carried out by aligning their developmental and ripening stages. As shown by Klie *et al.* (2014), the climacteric (peach and tomato) and non-climacteric (pepper and strawberry) species were found, using the computational program STASIS, to differ in their sugar and amino acid metabolism. Similarly, the work of Roch *et al.* (2019) compared the levels of primary metabolites in different fruits between development and ripening. There are myriad differences between them, but also some similarities, such as those in sugar levels, and we can classify them into hexose accumulators (raspberry, blackberry, kiwi, pepper, and eggplant), sucrose accumulators (melon, watermelon, and peach), or those that accumulate similar quantities of both (such as strawberry) (Roch *et al.*, 2019). Several important conclusions could be made from these studies as one of the main metabolic differences between climacteric and non-climacteric fruit is the use of aspartate and methionine for ethylene biosynthesis as well as malate (the aspartate precursor) as regulator of ethylene feedback (Osorio *et al.*, 2011), while in non-climacteric fruit, citrate is the major organic acid accumulated during this process. In general terms, although with some exceptions, climacteric fruits tend to accumulate higher concentrations of fructose and glucose than non-climacteric fruits (Fig. 1). On the other hand, non-climacteric fruits accumulate higher levels of sucrose in the early stages of development, which in turn is needed to trigger ripening (Durán-Soria *et al.*, 2020). In future studies it will be interesting to see the effect of minimizing availability of sugars and acids in climacteric fruit as well as evaluating the evolution of this trait.

Thus far we have largely discussed the use of metabolomics to study development up to ripening. Two further studies that look at post-harvest metabolism warrant mention here. The first of these detailed the identification of the *delayed fruit deterioration* (*dfd*) mutant (Saladié *et al.*, 2007). This mutant, which is actually likely a heirloom variety, is characterized by partial ripening based on a number of canonical ripening-related traits, yet whole-fruit firmness is prolonged for many months after the onset of ripening (Saladié *et al.*, 2007). A comparative GC-MS-based study of on and off the vein post-harvest characteristics of *nor*, *rin*, and *dfd* and the wild type cultivars Alicia Craig and M82 revealed that *dfd* resulted in prolonged firmness without compromising key quality metabolites (see below), highlighting the importance of putrescine in extending shelf-life (Osorio *et al.*, 2020). The comparison between on and off the vine ripening, meanwhile, provided insights into the significance of the import of metabolites such as sucrose during ripening.

In summary, the application of metabolomics has provided us with a far more sophisticated understanding of the metabolic shifts intertwined with fruit ripening and development. Before finishing this section, it would be remiss not to discuss recent findings regarding the roles of the classical ripening-regulated proteins. Giovannoni *et al.* (2017) and Ito *et al.* (2017) recently revealed by the use of CRISPR gene editing that the *rin* allele encodes a dominant repressor, not activator, of ripening that operates by binding many of the promoters that RIN normally activates. However, it is important to note the argument of Wang *et al.* (2020) that the fact that CRISPR mutants result in unexpectedly weak phenotypes suggests the presence of compensatory mechanisms that may obscure gene function. It therefore seems prudent to re-examine this phenomenon as further examples emerge.

### Use of metabolomics to aid efforts in improving fleshy fruit taste

As for ripening, a large portion of the research looking into improving fruit taste has focused on tomato. Given that this has been the subject of several excellent recent reviews (Klee and Tieman, 2018; Zhu *et al.*, 2019; Wang *et al.*, 2023), we will not cover this area in as much detail as that of ripening. However, it is important to note that consumer demand has in recent years somewhat changed the target of breeders—a fact that is easily observable by the fact that consumers are happy to pay a premium for better tasting foods. The taste of fruits is largely down to a relatively small subset of the metabolome encompassing sugars and organic acids, the umami metabolite glutamate, and the several hundred volatile organic compounds that tomato fruit have been characterized as emitting. Whilst not wanting to re-iterate what is covered in earlier reviews, we feel that three different approaches to using metabolomics to investigate aspects of tomato fruit taste merit discussion here, namely (i) the use of quantitative genetic metabolomics to determine the genetic factors contributing to the accumulation of metabolites known or at least thought to be important contributors to taste, (ii) the use of taste panels to assess which compounds are important contributors to taste, and (iii) the use in very recent studies of machine learning models to predict how flavorful a fruit will be on the basis of its chemistry.

As we mentioned above, the contents of the major sugars, sucrose, glucose, and fructose (Carrari *et al.*, 2006), the major organic acids, citrate and malate (Centeno *et al.*, 2011), and the umami conferring amino acid glutamate (Grierson *et al.*, 1985) alongside the 400-odd volatile organic compounds synthesized by tomato (Buttery *et al.*, 1989) represent the main contributors to tomato taste. Both QTL and more recently GWAS studies have led to the identification of the genomic regions, or indeed the genes, that control the level of accumulation of many of these metabolites (Fridman *et al.*, 2004; Tieman *et al.*, 2017; Ye *et al.*, 2017; Bulut *et al.*, 2023). Recently, CRISPR–Cas9 was used to edit the invertase inhibitor gene *SlNVIH1* in tomato. The edited plants displayed higher solid soluble content as well as higher sugar levels (Suc, Glc, and Fru) than the non-edited plants without a reduction in fruit weight as a tradeoff. (Kawaguchi *et al.*, 2021). Whilst not directly evaluating the genes that determined the metabolite content, a recent multiomics study into the rewiring of the fruit metabolome during breeding (Zhu *et al.*, 2018) is highly interesting in this context. This study revealed that the selection for alleles of genes associated with larger fruits altered metabolite profiles as a consequence of linkage to nearby genes, that breeding for the Asian preferred pink tomato modified some 100 metabolites, and that the introgression of resistance genes also affected a wide range of metabolites.

The studies described above have yielded a number of tools that would allow targeted breeding for taste. That said, despite the monetary and labor costs involved, studies that pair metabolomics with taste-panel tests will likely yield superior information given that taste is very much a composite trait (Tikunov *et al.*, 2013; Klee and Tieman, 2018). In this vein, in the groundbreaking study of Tieman *et al.* (2012), metabolomics and tasting panels were used to produce a predictive and testable model for liking. A more recent study demonstrated that machine learning can predict consumer ratings of liking, sweet, sour, umami, and flavor intensity thereby greatly reducing both the monetary and the labor input to such studies (Colantonio *et al.*, 2022). Indeed, adoption of such approaches will greatly enhance the throughput of flavor evaluations likely rendering it possible to adopt them into breeding pipelines and hopefully ultimately allowing the design of varieties with exceptional flavor properties (Colantonio *et al.*, 2022; Fernie and Alseekh, 2022a).

### Use of metabolomics to aid efforts in improving nutritional quality of fleshy fruits

Attempts to improve nutritional quality largely follow similar methods to those described above for improving taste; the target molecules are normally quite different, though, with most of the targets for nutrition being plant specialized metabolites. As for taste, nutritional improvement of tomato has been well reviewed recently (Li *et al.*, 2018), so we will largely focus on aspects in which metabolomics has played a key role. Like taste, nutrition is often complicated by the presence of multiple metabolites in a foodstuff, and hence ultimately following the production of putatively more nutritious food it is essential that any potential health benefits are adequately assessed. The study of Zhu *et al.* (2018) mentioned above identified five genetic loci responsible for the down-regulation of anti-nutrient solanaceous glycoalkaloids during the domestication of tomato. Similarly, QTLs for these compounds as well as potentially nutritious flavonoids were identified in the evaluation of the *Solanum pennellii* introgression lines (Alseekh *et al.*, 2015). Metabolomics was also used in support of recent metabolic engineering strategies that generated transgenic lines exhibiting elevated levels of anthocyanins (Butelli *et al.*, 2008; Tohge *et al.*, 2015; Zhang *et al.*, 2015), l-Dopa (Breitel *et al.*, 2021), and vitamin D (Li *et al.*, 2022). These studies helped establish the underlying pathways as well as other metabolic changes that they invoked, providing us with greater prospects to develop more nutritious crops. Whilst many of these approaches involved transgenic approaches, enhancing vitamin D was achieved via gene editing, which is allowed in an increasing number of countries worldwide.

## Berries and receptacle fruits

By contrast to fleshy fruits, both berries and receptacle fruits (described here) as well as stone fruits (described in the next section) have received considerably less research attention. For this reason, we will not sub-divide the research on the basis of research approach but rather discuss them as one. Berries, including strawberries, are top of consumers’ preference on account of their remarkable taste, their vibrant colors, and their enticing aromas. These fruits also offer a plethora of health benefits due to their high content of vitamins, minerals, and phytochemicals (Giampieri *et al.*, 2015). The organoleptic properties of berries are vital factors contributing to their elevated value and widespread consumer appeal, and therefore metabolic research in these fruits is gaining traction. There are many studies that have identified the metabolic composition of these fruits, such as blackberry (Wu *et al.*, 2023), raspberry (Paudel *et al.*, 2013), strawberry (Fait *et al.*, 2008), and blueberry (Xie *et al.*, 2022), or have carried out flavonoid profiling in a range of different berries (Ponder *et al.*, 2021). However, tracing the genes responsible for the variation in metabolic composition or identifying new molecular markers for breeding remains difficult due to the fact that berries have complex genomes, such as cultivated strawberry, which has an octoploid genome (Edger *et al.*, 2019), or blueberry, which is tetraploid (Colle *et al.*, 2019). As a result, these have only been sequenced in the last 5 years, while others such as raspberry (VanBuren *et al.*, 2016) were sequenced relatively early and yet others still do not yet have a complete genome. This means that as yet there are very few GWAS studies on berry metabolites. Nonetheless, within the Goodberry European project, it seems reasonable to anticipate that the provisions for new genomic, transcriptomic, and metabolomic data will greatly enhance our understanding of these relatively poorly studied species (Senger *et al.*, 2022).

As strawberry is the most extensively studied member of its family, we will focus on the metabolomic changes recorded in this fruit. Early studies revealed the main differences in the metabolomic composition of cultivated and wild strawberries (Aharoni *et al.*, 2004; Ulrich *et al.*, 2007). Such species as *Fragaria vesca*, *Fragaria virginiana*, and *Fragaria moschata* present a diverse flavor that is not matched by commercial *Fragaria* × *ananassa* varieties. Cultivated strawberries are sweeter and present a more balanced sugar/acid taste, while wild ones present a variety of aromas that are not present in the cultivated, but in turn, lack the taste and mouth sensation that consumers prefer (Ulrich *et al.*, 2007; Fait *et al.*, 2008). Sensorial aspects have been largely ignored in breeding programs in favor of other aspects such as yield and size (Qin *et al.*, 2008). Species such as *F. moschata* and accessions of *F. vesca* display higher levels and more diverse composition of esters than in cultivated strawberry, such as methyl cinnamate and methyl anthranilate, found exclusively in diploid strawberry, and terpenes, which give a fruity aroma (Ulrich *et al.*, 2007). Other compounds have been lost during strawberry domestication; some monoterpenes are present in diploid species and not in octoploid ones, such as α-pinene, β-myrcene, and myrtenol (Aharoni *et al.*, 2004). This metabolic loss is due to mutated alleles of a *pinene synthase* being present in *F. ananassa* while functional alleles are present in the diploid *F. vesca* (Aharoni *et al.*, 2004). In contrast, the synthesis of linalool, one of the main compounds of strawberry aroma, follows a distinct pattern of presence between octoploid cultivated and the diploid species. This divergence is attributed to a specific mutation in the enzyme *nerolidol synthase 1* that produced a shift in the subcellular localization from the cytosol in the producing varieties to the chloroplast in the non-producing ones. Consequently, this alteration prompts the linalool non-producing varieties to generate an alternative compound, nerolidol, which is found in diploid *F. vesca* (Aharoni *et al.*, 2004; Folta and Klee, 2016).

One of the main differences between diploid *F. vesca* and octoploid *F. ananassa* is the skin or flesh color. While the most studied varieties of *F. vesca* display both a red skin and white flesh, the majority of the *F. ananassa* accessions present colored skin and flesh (Sun *et al.*, 2014). These differences are due to a mutation in the *F. vesca MYB10* gene, encoding a TF that regulates the anthocyanin biosynthesis pathway (Medina-Puche *et al.*, 2014; Hawkins *et al.*, 2016). A more detailed study of diverse octoploid varieties with contrasting skin or flesh coloration revealed that the color variance was caused by independent mutations in the *FaMYB10* gene (Castillejo *et al.*, 2020).

Despite the above-mentioned genomic complexity of strawberry, there are some example of GWASs on primary, secondary, and volatile compounds that have led to the identification of QTLs and genes of interest for agricultural breeding, such as a QTL associated to the levels of the volatile γ-decalactone (Sánchez-Sevilla *et al.*, 2014; Cruz-Rus *et al.*, 2017). This study was followed up by the publication of a PCR protocol to rapidly identify the gene (Cruz-Rus *et al.*, 2017).

Recently, Fan *et al.* (2022) described several expression QTLs related with volatile content in a panel of 305 different strawberry accessions. Some of these correspond to individual genes that were previously known to affect different compounds (some of which will be covered in the next section) as well as up to 14 novel candidate genes that could be used for breeders not only in strawberry but in other fruits that share a common volatilome with strawberry such as apples, peaches, and oranges. Using an F_1_ population between two parents with contrasting sugar and acid levels, Vallarino *et al.* (2019) identified mQTLs related with primary metabolism using a GC-MS platform, allowing them to identify a region associated with sugar content on chromosome 5 and another with acid and pH of the fruit in the same region. Moreover, three QTLs were detected related with vitamin C content, with one of them later being identified by Muñoz *et al.* (2023) using a different population. Indeed, in the latter study expression of the identified candidate genes was found to correlate with ascorbic acid levels, making them good candidates for targeted breeding. In these studies, the different metabolite identification platform allowed the researchers to find unknown genes linked to the content of several metabolites that could be potentially used as targets in breeding programs.

As for tomato, early studies using a GC-MS platform characterized the dynamics of sugar, acid, and amino acid contents along the course of fruit ripening in strawberry (Fait *et al.*, 2008; Zhang *et al.*, 2011). Given the peculiarity that strawberry is a receptacle with the real fruit embedded on the surface, similar studies have been performed in the achenes (Fait *et al.*, 2008). Along the course of ripening, the strawberry receptacle accumulates high levels of sugars (Suc, Fru, Glc, and Rib), with sucrose accumulation being necessary to trigger ripening (Jia *et al.*, 2013), and organic acids such as malate and citrate (Fait *et al.*, 2008). Nonetheless, in contrast to the situation observed in tomato, in strawberry, TCA cycle intermediates do not increase in level during development and ripening; rather, they display high levels across the process, revealing that the sugar/acid ratio is due rather to changes in sugar dynamics (Fait *et al.*, 2008; Zhang *et al.*, 2011; Ornelas-Paz *et al.*, 2013). Although the majority of studies are focused on the receptacle, Fait *et al.* (2008) also described the changes produced on the achene, which displayed a sharp reduction in both sugars and organic acids during ripening.

One of the families of compounds that have been extensively investigated in strawberry fruits is the anthocyanins. Responsible for characteristic red or violet color, these compounds also contribute to sensorial and health properties of several berries (Krga and Milenkovic, 2019). As in the case with sugars, through LC-MS studies a great increase of anthocyanin and flavonoid levels has been found in strawberry with pelargonidin and cyanidin-3-glucose being the major compounds accumulated (Aaby *et al.*, 2007), while proanthocyanins are accumulated in the green stages, probably to protect the developing fruit from animals, given their astringent taste (Buendía *et al.*, 2010). This accumulation of compounds is additionally in close accordance with the changes in the transcriptome during ripening (Baldi *et al.*, 2018).

The study of natural mutants has led to the elucidation of the genetics underlying white fruit mutants. For example, a 5 bp insertion in *anthocyanidin synthase* of a natural yellow raspberry caused this phenotype (Rafique *et al.*, 2016), while a white bilberry mutant displayed lower activities of the structural enzymes dihydroflavonol 4-reductase and flavanone 3-dioxygenase (Zorenc *et al.*, 2017). In mulberry, the loss of the TF bHLH3 is related to the decrease of anthocyanin accumulation, resulting in a redirection of the carbon flux into proanthocyanins, thereby generating pale fruits (Li *et al.*, 2020). Several mQTL controlling flavonoids were identified by Urrutia *et al.* (2016) using a near isogenic line of the diploid *F. vesca*. The identified regions contained genes of known function such as *dihidroflavonol reductase* and *flavonol synthase* (Almeida *et al.*, 2007), *flavonoid 3ʹhydroxylase* (Thill *et al.*, 2013), and two glucosyltransferases (Lunkenbein *et al.*, 2006; Griesser *et al.*, 2008). Other QTL regions were later confirmed using octoploid strawberry cultivars. For example, a region related to pelargonidin-3-*O-*malonylglucoside biosynthesis, which was defined by Davik *et al.* (2020), was previously found by Urrutia *et al.* (2016). These malonyltrasferases are transcriptionally controlled by a MYB TF named FaMYB123, which plays a specific role controlling the late steps of the anthocyanin biosynthesis (Martínez-Rivas *et al.*, 2023). In these studies, using the LC-MS platform, the authors were able to follow the carbon flux when the genes were down-regulated, as a proof of their biological function. Regarding the control of anthocyanin, it is worth mentioning that *MYB10* has been described as a master regulator of anthocyanin and flavonoid accumulation in various fruit species (Lin-Wang *et al.*, 2010; Medina-Puche *et al.*, 2014). Transcription factor genes identified by RNAi experiments also resulted in white fruits such as *ripening induced factor* (*FaRIF*) (Martín-Pizarro *et al.*, 2021) or *related to ABI3/viviparous 1* (*FaRAV*) (Zhang *et al.*, 2020). However, a more detailed review of the TFs controlling strawberry ripening was recently published by Sánchez-Gómez *et al.* (2022), and we refer readers to this for further details.

As mentioned above, the genomic complexity of berries rendered it difficult to ascertain the function of single genes and their impact on metabolic changes. Nonetheless, enzymes pertaining to the phenylpropanoid pathway have been extensively studied in several species, for example strawberry (Lunkenbein *et al.*, 2006; Almeida *et al.*, 2007; Griesser *et al.*, 2008), blackberry (Xie *et al.*, 2022), and blueberry (Chen *et al.*, 2012) to name but a few. Multiple enzymes related with aroma have been characterized in diverse *Fragaria* such as the alcohol acetyl transferases (Beekwilder *et al.*, 2004; González *et al.*, 2009; Cumplido-Laso *et al.*, 2012), quinone reductase (Raab *et al.*, 2006) or carboxylesterase (Martínez-Rivas *et al.*, 2022), all of which are involved in production of key compounds such as esters and furaneol that accumulate on ripening.

Though there are several studies that have focused on the health-related benefits of ingesting different berries, as raw fruits, juices, or in multiple preparations (Cassidy, 2018; Ho *et al.*, 2020), we still lack information on how to improve the nutritional quality of these species. Our understanding of berry metabolism has recently greatly increased. As such we are in a far better position to influence the levels of metabolites in these species. Given the fact that successful transformation protocols exist for many of these species along with the recent boom in horticultural research (Jiang *et al.*, 2022), it seems reasonable to anticipate that our knowledge gain will accelerate in the next few years. It thus seems viable that biofortification of fruits could represent a tangible and important future breeding goal.

## Stone bearing fruits

Stone bearing fruits such as plums, apricots, and peaches produce a lignified endocarp that protects the seed, with a mesocarp rich in minerals, vitamins, and other health promoting compounds (Dardick and Callahan, 2014; Lara *et al.*, 2020). These species are considerably less studied in the metabolomics field. However, there are still a handful of studies worth discussing. Like berries, the stone bearing fruits have a sweet taste that is one of the most appreciated features for consumers (Delgado *et al.*, 2013). Similarly, GC-MS-based measurements revealed that peach accumulates high levels of sugars (Suc, Glc, and Fru) during the ripening process. Malate does not accumulate but rather maintains high levels (as in strawberry), while citrate levels increase (differing from strawberry). These increases of metabolite levels were accompanied by a study on enzymatic activity or gene expression that supported the metabolomic findings, with, for example, a higher expression of sucrose synthase and sugar transporters being found (Lombardo *et al.*, 2011).

As in berries, the color compounds are among the most studied. Different peach varieties produce red mesocarp if accumulating anthocyanins and yellow mesocarp if accumulating carotenoids, while white peaches do not accumulate high levels of either pigment (Cantín *et al.*, 2009). Consumers prefer yellow peaches in Western countries but white varieties in Eastern countries (Song *et al.*, 2022). As discovered by analysing 35 genotypes, ancestral species of peach display white flesh, and during domestication, a mutation in a *carotenoid cleavage dioxygenase* resulted in yellow-flesh peaches with different flesh color. Furthermore, an F_1_ population of a white × yellow cross revealed that down-regulation of the *9-cis-epoxycarotenoid dioxygenase* gene also produced yellow traits (Song *et al.*, 2022). White and yellow peaches do not differ only in flesh color, but also display a different aroma repertoire. Among the common compounds, white peaches accumulate more benzyl alcohol, 3-octanone and styrene, compounds with fruity and sweet aromas, while yellow fruits accumulate octanal and nonanal, giving a fruity aroma. They also displayed unique compound depending on the genotype, with the white genotype having notes of balsamic and fusel-like odor and the yellow a sweeter, citrus–banana-like odor (Liu *et al.*, 2022).

The study of 252 accessions of peaches revealed that during domestication a decrease in malate and citrate was selected, which may result in a monotonous taste (Cao *et al.*, 2022), most probably a consequence of selection by humans favoring yield over aroma and taste (Klee and Tieman, 2018). As mentioned above for strawberry, volatile compounds measured by GC-MS decreased in level and variety during domestication (Cao *et al.*, 2021). Among these compounds, three aldehydes (hexanal, (E)-2-hexenal, and benzaldehyde) and two lactones (i.e. nonalactone and δ-tetradecalactone) considerably decreased in level. These compounds are tightly related to the ‘peachy’ aroma sensed by humans (Wang *et al.*, 2009; Eduardo *et al.*, 2010). These types of studies open the possibility of improving modern cultivars using the wild ones to reintroduce the lost metabolites, with the help of metabolomic technology (Cao *et al.*, 2021, 2022).

Given that stone bearing fruits are less studied than berries or other species, more research effort in species such as cherries, apricots and nectarines is needed to decipher the metabolic qualities of these fruits. The studies in one species could help to improve the qualities of another as there are several examples of aroma related enzymes that present the same activity in different fruits. For example, alcohol acetyl transferases in strawberry (Beekwilder *et al.*, 2004) and in peach (Zhou *et al.*, 2021) present conserved domains though not that much similarity in the sequence. Moreover, they present similar activities towards the alcohol substrate, a case similar to that of the carboxylesterase enzymes that in both peach (Cao *et al.*, 2019) and strawberry (Martínez-Rivas *et al.*, 2022) have increased activity towards longer ester chain substrates. That said, considerably more studies on the metabolism in various fruit species are needed to assess similarities and differences between them.

## Conclusions and future perspectives

**Fig. 2.**
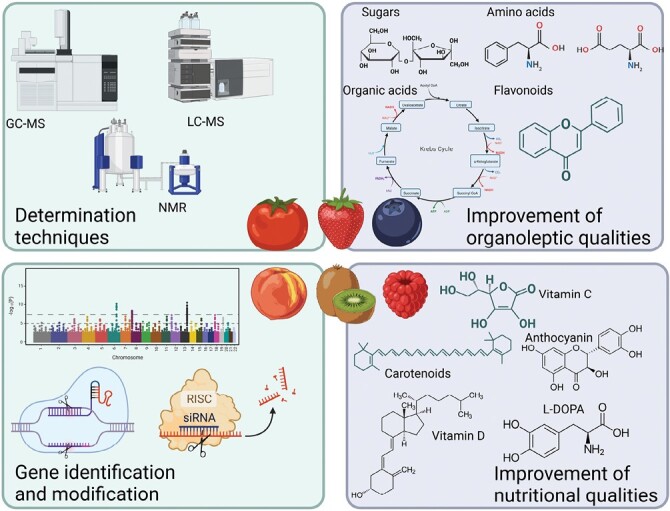
A schematic representation of how metabolomics can be applied to the improvement of different ripening-related characteristics. Created with BioRender.com.

The power of metabolomics to identify and improve biochemical pathways among species is potentially even greater than that of transcriptomics, as metabolites are not species specific. As stated earlier, the availability of multiple metabolomic studies in different species and tissues (climacteric versus non-climacteric, different treatments, response to multiple stresses, cultivation techniques, and so on) is opening a new window to multiple comparisons, and new tools such as STATIS are emerging. More recently the study of Colantonio *et al.* (2022) used machine learning to predict taste of tomato and blueberry on the basis of metabolic profiles. This approach is likely to revolutionize breeding in that it very much reduces the cost of metabolomic analysis, rendering it economically viable for breeding as it obviates the need for the more expensive expert panels for determining taste. As such it will greatly accelerate metabolomics-assisted breeding and arguably even finally render it economically viable. Whilst this will be of great value from an applied perspective, it may also accelerate our ability to obtain mechanistic insight into the control of metabolism. One of the main disadvantages of metabolomics is the identification of compounds. To improve it, it is important that good practices are used when reporting metabolomic data. For example, the construction of new databases is needed in order to collect all published data. It thus seems likely that the combination of metabolomics with other post-genomic technologies as well as with computational methods will greatly expand our understanding of and ability to influence fruit metabolism (Fig. 2).
